# Automatic detection of vessel structure by deep learning using intravascular ultrasound images of the coronary arteries

**DOI:** 10.1371/journal.pone.0255577

**Published:** 2021-08-05

**Authors:** Hiroki Shinohara, Satoshi Kodera, Kota Ninomiya, Mitsuhiko Nakamoto, Susumu Katsushika, Akihito Saito, Shun Minatsuki, Hironobu Kikuchi, Arihiro Kiyosue, Yasutomi Higashikuni, Norifumi Takeda, Katsuhito Fujiu, Jiro Ando, Hiroshi Akazawa, Hiroyuki Morita, Issei Komuro

**Affiliations:** 1 Department of Cardiovascular Medicine, The University of Tokyo Hospital, Tokyo, Japan; 2 Department of Advanced Cardiology, The University of Tokyo, Tokyo, Japan; Baylor Scott and White, Texas A&M College of Medicine, UNITED STATES

## Abstract

Intravascular ultrasound (IVUS) is a diagnostic modality used during percutaneous coronary intervention. However, specialist skills are required to interpret IVUS images. To address this issue, we developed a new artificial intelligence (AI) program that categorizes vessel components, including calcification and stents, seen in IVUS images of complex lesions. When developing our AI using U-Net, IVUS images were taken from patients with angina pectoris and were manually segmented into the following categories: lumen area, medial plus plaque area, calcification, and stent. To evaluate our AI’s performance, we calculated the classification accuracy of vessel components in IVUS images of vessels with clinically significantly narrowed lumina (< 4 mm^2^) and those with severe calcification. Additionally, we assessed the correlation between lumen areas in manually-labeled ground truth images and those in AI-predicted images, the mean intersection over union (IoU) of a test set, and the recall score for detecting stent struts in each IVUS image in which a stent was present in the test set. Among 3738 labeled images, 323 were randomly selected for use as a test set. The remaining 3415 images were used for training. The classification accuracies for vessels with significantly narrowed lumina and those with severe calcification were 0.97 and 0.98, respectively. Additionally, there was a significant correlation in the lumen area between the ground truth images and the predicted images (ρ = 0.97, R^2^ = 0.97, p < 0.001). However, the mean IoU of the test set was 0.66 and the recall score for detecting stent struts was 0.64. Our AI program accurately classified vessels requiring treatment and vessel components, except for stents in IVUS images of complex lesions. AI may be a powerful tool for assisting in the interpretation of IVUS imaging and could promote the popularization of IVUS-guided percutaneous coronary intervention in a clinical setting.

## Introduction

Cardiovascular disease due to atherosclerosis is the leading cause of death worldwide [1]. Percutaneous coronary intervention (PCI) is one of the therapeutic strategies for cardiovascular disease, and intravascular ultrasound (IVUS) can be performed to examine the components of vessels with atheromatous plaques pertaining to cardiovascular disease [2]. Therefore, in a clinical setting, pre-intervention IVUS should be useful to examine the target lesion and choose the optimal treatment strategy.

The Providing Regional Observations to Study Predictors of Events in the Coronary Tree (PROSPECT) study, which used IVUS to assess the lesion-related risk for major adverse cardiac events (MACEs), showed that non-culprit vessels with a lumen area less than 4 mm^2^ had a higher risk for future MACEs than those with a larger area [3]. Another study, which regarded the minimum lumen area of the unprotected left main coronary artery assessed by IVUS as an index for whether to perform PCI, reported that IVUS was more accurate than angiographic measurement for evaluating the extent of stenosis of target lesions [4]. IVUS is useful for choosing the optimized stent implantation for a target lesion and IVUS-guided PCI has several other advantages [5, 6]. However, pre-intervention IVUS is not widely performed worldwide [7, 8], partly because of the substantial knowledge and experience required for the interpretation of IVUS images.

In recent years, reports have shown that artificial intelligence (AI) can help improve the efficiency of medical care [9, 10]. Semantic segmentation is one task in the realm of AI-performed object recognition, and involves the categorization of every pixel in an image to one of several classes or concepts [11]. Although previous studies reported the segmentation of IVUS images, this segmentation was performed only for the lumen and media [12, 13]. Moreover, segmentation was not performed on IVUS images of complex lesions containing severe calcification or stenosis [14].

To optimize stent implantation using IVUS imaging guidance, the degree of calcification of vessels in complex lesions must be evaluated, as well as their lumen area. Severely calcified lesions have a higher risk of re-stenosis than less severely calcified lesions [15]. Therefore, evaluating the location and the arc of calcification for the target lesion in IVUS images is essential for preventing complications in PCI procedures and improving the clinical outcome after PCI [2, 16]. If AI could categorize vessel components in IVUS imaging and suggest the optimal PCI strategies to cardiologists, more efficient and safer PCI could be performed. However, whether AI can categorize vessel components, including calcification, has not been reported. In the present study, we describe development and evaluation of a new AI program that can classify vessels with significantly narrowed lumina and those with severe calcification. This AI program can also categorize vessel components and stents, even in IVUS images of complex lesions.

## Materials and methods

### Patients

Patients with stable angina pectoris who underwent pre-intervention IVUS imaging assessment of the culprit vessel at The University of Tokyo Hospital between January and March 2019 were retrospectively selected. We included patients whose pre-intervention IVUS imaging assessment was made using a high-definition 60-MHz IVUS system (AltaView, Terumo, Tokyo, Japan). We excluded patients who underwent pre-balloon dilation or use of a debulking device before IVUS assessment, and those with poor-quality IVUS images. Finally, we analyzed IVUS imaging data from 24 patients.

This study was approved by the Institutional Review Board of The University of Tokyo (approval number 2650). The requirement for individual informed consent was waived.

#### Coronary angiographic analysis

Quantitative coronary angiography software (QAngio XA 7.3, Medis Medical Imaging Systems BV, Leiden, The Netherlands) was used to analyze coronary lesions. The parameters that were measured included lesion length and minimum lumen diameter [17]. The American Heart Association/American College of Cardiology classification system was used to evaluate lesion complexity and type B2 and C lesions were considered complex [18].

### IVUS image acquisition

IVUS examinations were performed using a high-definition 60-MHz IVUS system (AltaView) and imaging data were analyzed with a Visicube IVUS imaging system (Terumo). All images were collected at a rate of 3 mm s^-1^ using an automatic pullback system.

### Preparation of IVUS image sets

**Fig 1:**
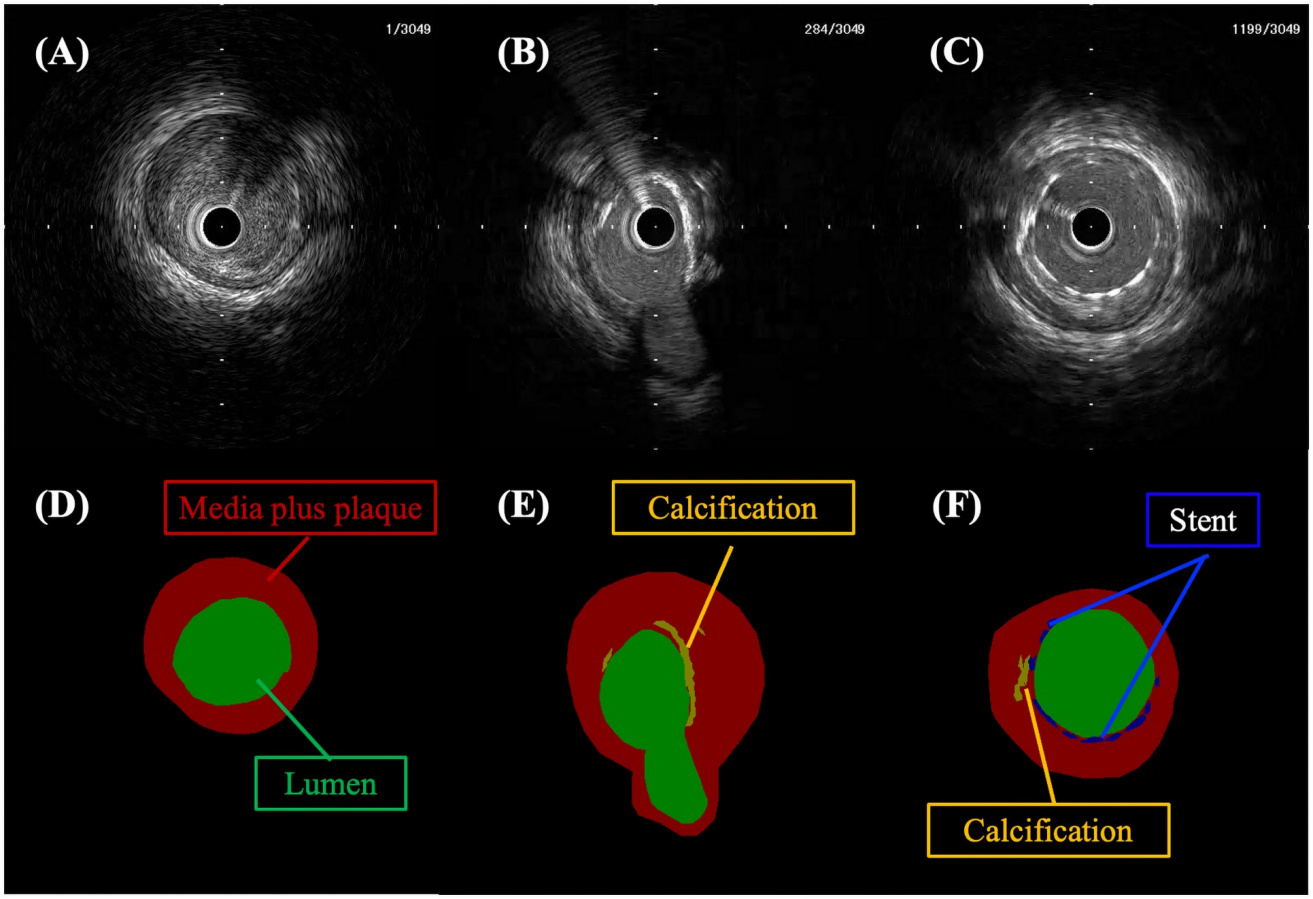
Representative manually segmented IVUS images and mask images. Upper images show IVUS images and lower images show mask images. Panel D is the mask image segmented from panel A. Similarly, panel E corresponds to panel B and panel F corresponds to panel C. The green, red, orange, and blue areas denote the lumen area, the medial plus plaque area, calcification, and a stent, respectively. The black areas show background.

Cross-sectional IVUS images were taken from the IVUS video and images were extracted at 0.5-mm intervals. First, manual segmentation to categorize the vessel components and stents into each label was performed by a single interventional cardiologist (H.S.) according to the following definitions, with use of the noncommercial software Labelme [19]. Each IVUS image was manually segmented into one of four classes, each with a unique color. Green was used for the lumen area, red for the medial plus plaque area, orange for calcification, and blue for a stent. Next, two more interventional cardiologists (Susumu K. and Satoshi K.) evaluated the images and labels, and disagreements were resolved by consensus. Consequently, mask images composed of these four classes were generated for the deep learning (Fig 1). Lumen areas were defined as areas surrounded by the luminal border [20]. Medial plus plaque areas were defined as areas surrounded by the leading edge of the adventitia minus the lumen area [20]. Stent struts were defined as points or arcs with high echogenicity along the circumference of the vessel [20]. Areas of calcification were defined as bright echo points or regions with an acoustic shadow [20]. Additionally, a lumen was considered significantly narrowed if it had an area less than 4 mm^2^ [3], while severe calcification was defined as calcification with an arc in more than two quadrants [21]. The remaining black areas, which had no manual annotation, were defined as background.

### Algorithms

**Fig 2:**
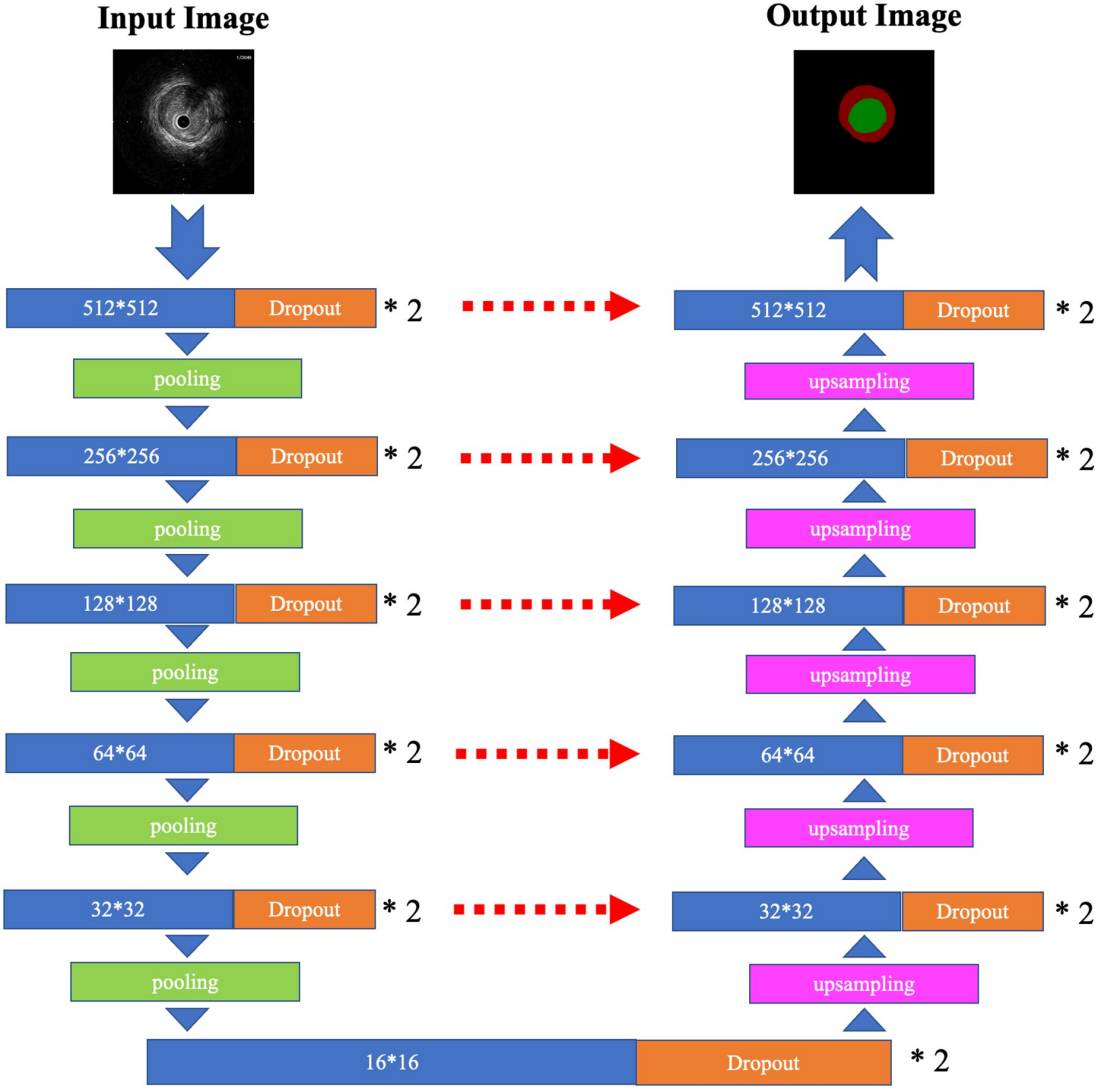
Illustration of U-Net used in this study. The architecture of U-Net used in this study is illustrated. The blue block and orange block are two-dimensional convolutional layers (kernel size = 3 × 3, stride = 1) with dropout (rate = 0.1). The number in the blue block denotes the feature map size. The green block shows the maximum pooling layer (pool size = 2 × 2), the pink block shows the upsampling layer (size = 2 × 2), and the red dashed arrows show the concatenation of the two layers.

To automatically recognize vessel components and a stent in the cross-sectional IVUS images, a deep neural network framework called U-Net was used [22] (Fig 2). The batch sizes, loss function, and optimizer were set at 2, categorical cross-entropy, and Adam (learning rate: 0.001), respectively. Horizontal and vertical flips and rotations were used for data augmentation, and 20% of the training sets were used for validation. A Xeon Platinum 8180 central processing unit and Tesla V100 graphics processing unit were used for calculations.

### Outcome measures and statistics

To evaluate the capabilities of our AI system, we calculated the accuracy, recall score, and precision score for the classification of vessels with significantly narrowed lumina (< 4 mm^2^) and those with severe calcification. The lumen area was calculated using OpenCV with Python.


$$Accuracy=\frac{TP+TN}{TP+FP+TN+FN}$$

$$\text{Recall}\;\text{score}=\frac{TP}{TP+FN}$$

$$\text{Pricision}\;\text{score}=\frac{TP}{TP+FP}$$

FN = false negative; FP = false positive; TN = true negative; TP = true positive.

We also evaluated the correlation between lumen areas in the manually-labeled ground truth images and those in the AI-predicted images. Additionally, the intersection over union (IoU) and Dice scores of each category in the test set were calculated as true positive, true negative, false positive, and false negative on the basis of consistency of the number of the pixels in the ground truth images and predicted images [12]. The IoU and Dice scores ranged from 0 to 1, with a higher number indicating a better segmentation result. We evaluated the mean IoU and mean Dice scores calculated from the average of the IoU and Dice scores in each class. We also calculated the recall score for detecting stent struts in each IVUS image with a stent in the test set to evaluate how well our AI could detect stents. Additionally, the IoU and the Dice score of the lumen area and the medial plus plaque area were evaluated when the five classes were divided into the two following groups: lumen area and other classes, and medial plus plaque area and other classes.

Categorical variables are presented as number (%) and continuous variables as mean (± standard deviation). Spearman’s rank correlation test was performed to evaluate the relationship between the predicted images and the ground truth images. All differences were evaluated at the 95% level of significance (p < 0.05) using Python.


$$IoU=\frac{TP}{TP+FP+FN}$$

$$Dice\;score=\frac{2TP}{2TP+FP+FN}$$

$$mean\;IoU=\frac{1}{n}\sum_{i=1}^{n}IoU_{i}$$

$$mean\;Dice\;score=\frac{1}{n}\sum_{i=1}^{n}Dice\;score_{i}$$

FN = false negative; FP = false positive; IoU = intersection over union; TP = true positive.

## Results

**Fig 3:**
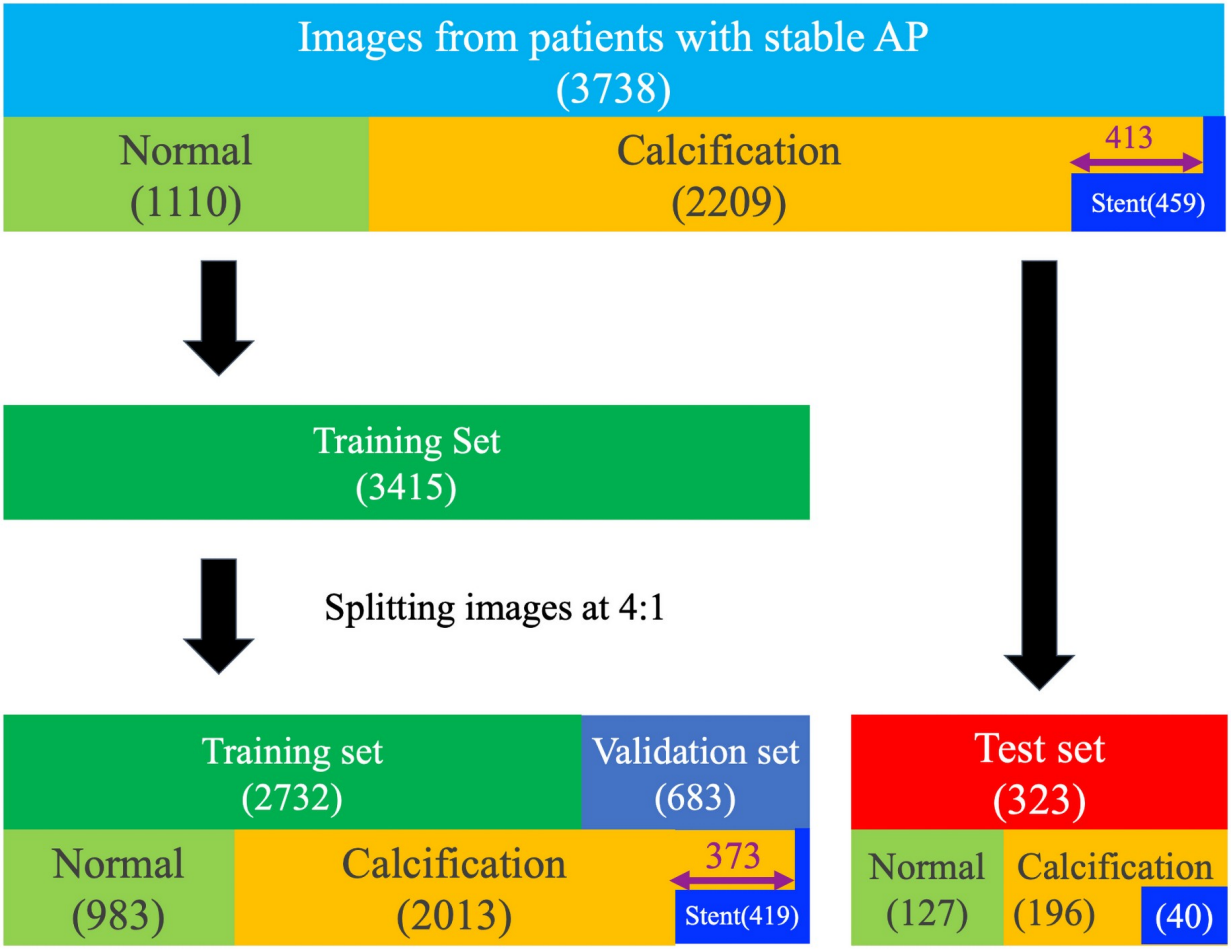
Study flowchart. “Normal” means that the images were without calcification or a stent. Of the 3738 IVUS images, 413 had both calcification and stents (purple double-headed arrow). In the test set, all 40 images with stents also had calcification. In the training set, 373 images with stents had calcification. AP = angina pectoris.

A total of 3738 IVUS images were labeled. Of these 3738 images, 2209 (59.1%) showed calcification and 459 (12.3%) showed a stent. A mean of 156 ± 50.3 IVUS images were extracted from one patient. Additionally, 635 of the 2209 images that showed calcification also showed severe calcification. Among the total IVUS images, 323 images from two patients were randomly selected and used for the test set. The remaining 3415 images from 22 patients were used for the training set, from which 683 images were used for validation (Fig 3). The data augmentation procedures, which are described above, were then used to increase the number of training set images to 103,837.

Coronary angiographic findings showed that the mean minimum lumen diameter and length of the lesions in the training set were 1.21 ± 0.48 mm and 46.7 ± 20.4 mm, respectively, whereas those in the test set were 0.63 ± 0.09 mm and 56.7 ± 17.9 mm, respectively. All lesions were complex lesions classified as B2 or C.

**Fig 4:**
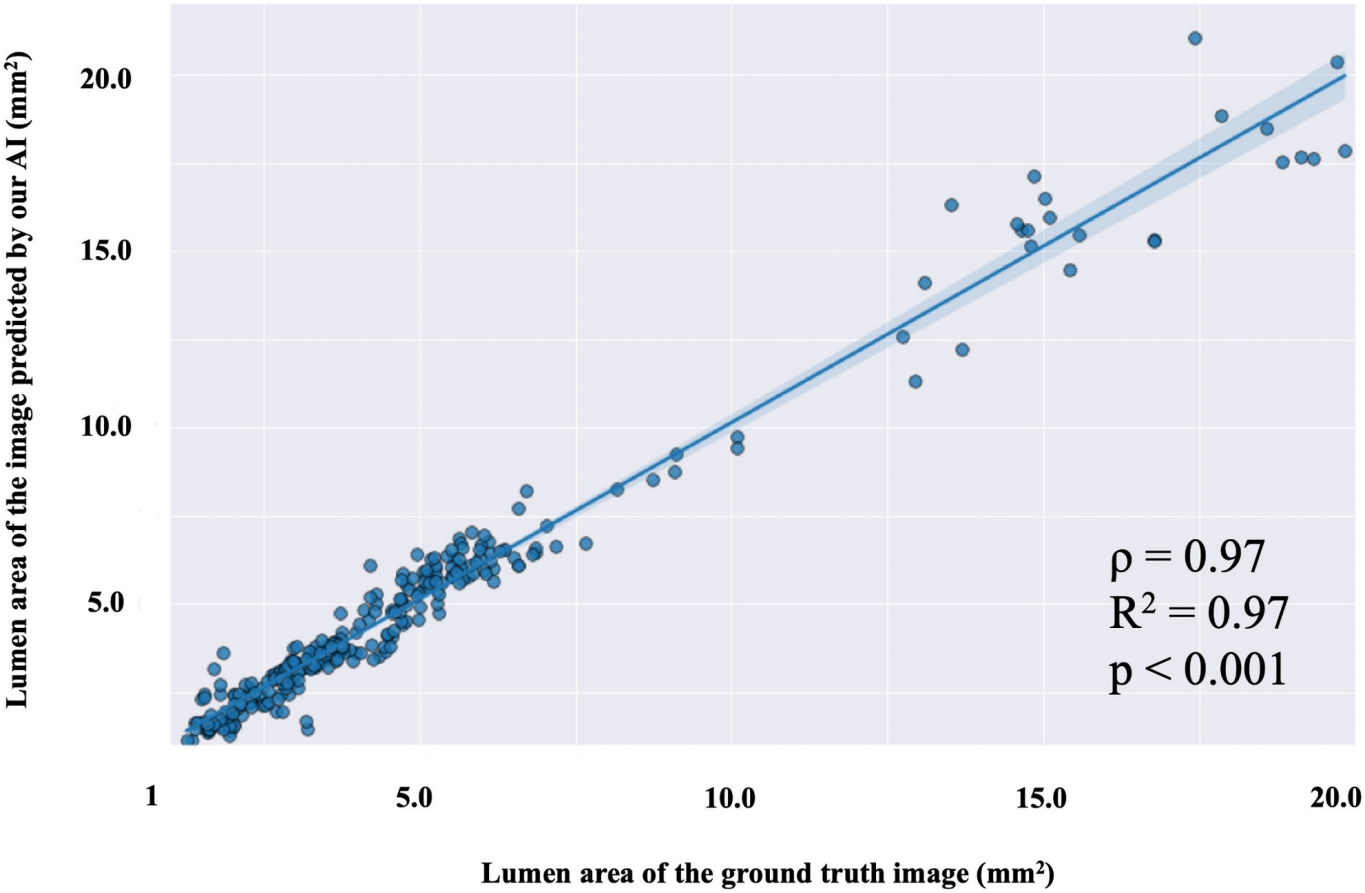
Scatter plot showing a regression line for the lumen area in ground truth mask images and the lumen area in mask images predicted by our AI. The light blue area shows the 95% confidence intervals of the regression line. Lumen areas of mask images predicted by our AI and lumen areas of ground truth mask images show a strong positive correlation (Spearman rank correlation).

**Fig 5:**
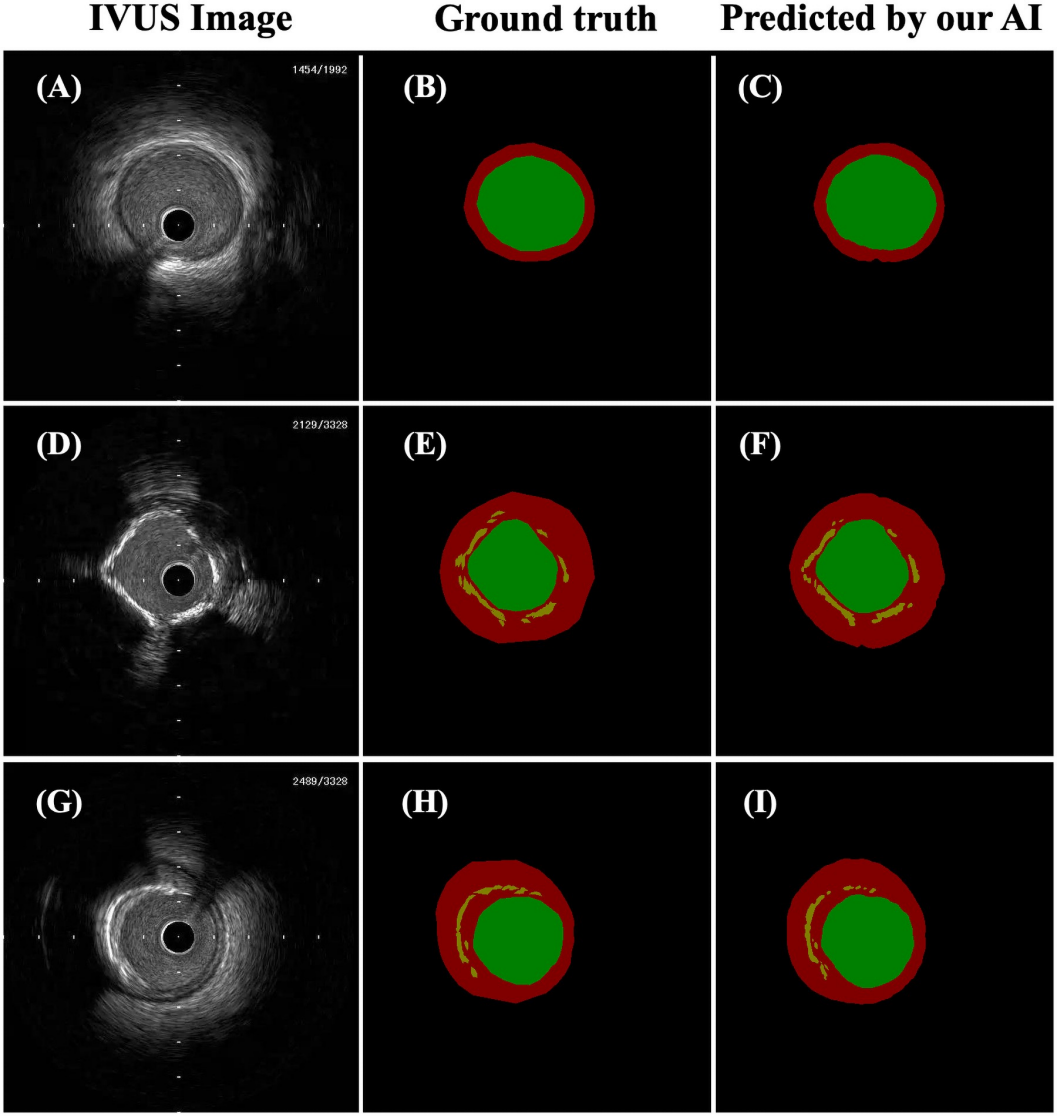
Representative images of cases successfully predicted by our AI. The panels on the left (A, D, G) show IVUS images. The middle panels (B, E, H) show ground truth mask images that were manually segmented corresponding to IVUS images on the left in the same row. The panels on the right (C, F, I) show mask images predicted by our AI corresponding to IVUS images on the left in the same row. In the mask images, the green area shows the lumen area, the red area shows the medial plus plaque area, the orange area shows calcification, and the black area is background. Calcifications in the IVUS images were effectively segmented by our AI (F, I).

**Fig 6:**
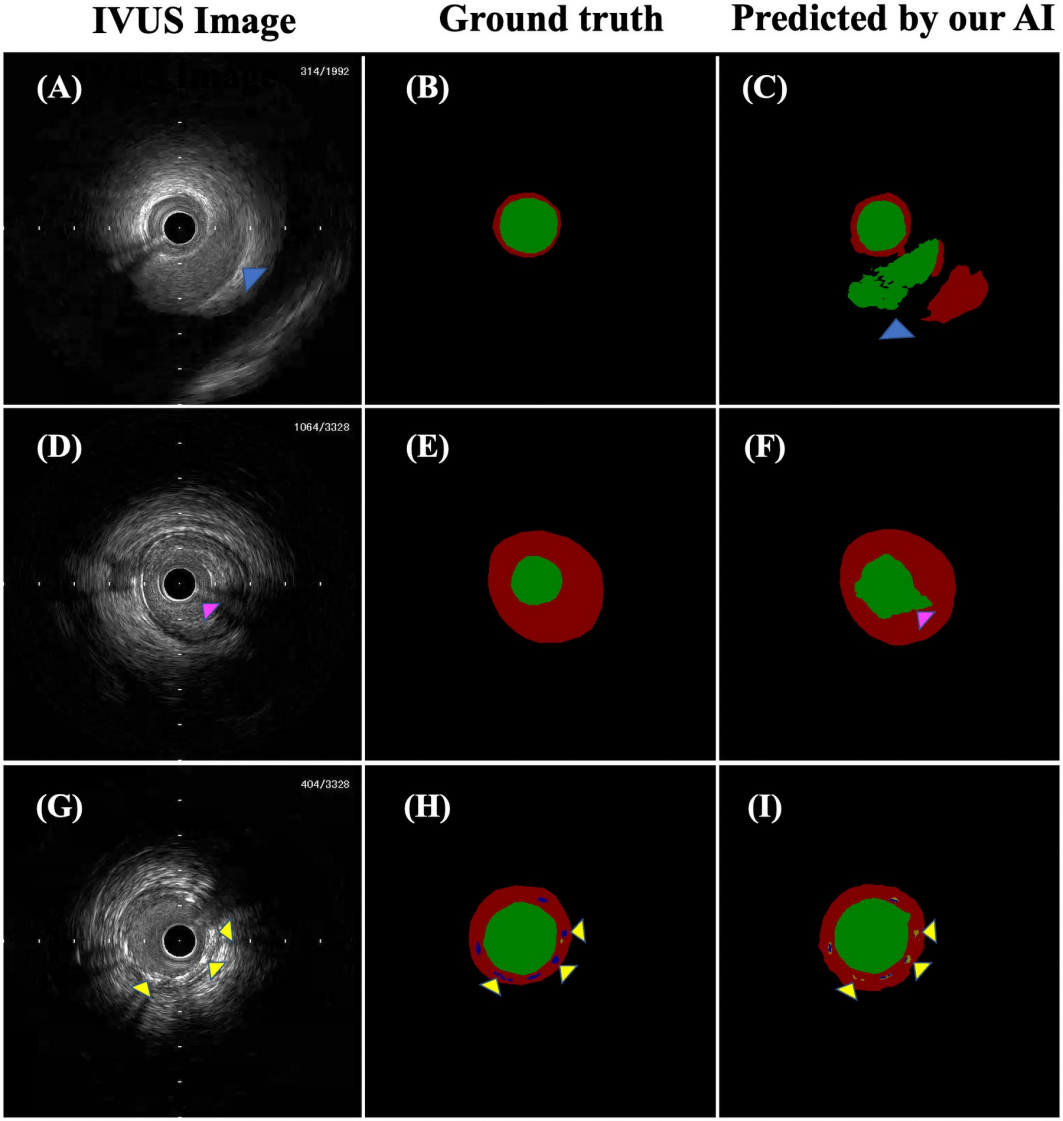
Representative images of cases showing prediction failure by our AI. The panels on the left (A, D, G) show IVUS images. The middle panels (B, E, H) show ground truth mask images that were manually segmented corresponding to IVUS images on the left in the same row. The panels on the right (C, F, I) show label images predicted by our AI corresponding to IVUS images on the left in the same row. In the mask images, the green area shows the lumen area, the red area shows the medial plus plaque area, the orange area shows calcification, the blue area shows a stent, and the black area is background. Two lumina were delineated in panel C because of misidentification of the coronary vein in panel A as the lumen of the coronary artery (blue arrowhead). The lumen edges in panel F were incorrectly delineated because of an artifact of the wire in panel D (pink arrowhead). The stent struts in panel I were misidentified as calcification (yellow arrowhead).

For the classification of vessels with a narrowed lumen area of less than 4 mm^2^ in IVUS images of the test set, the accuracy was 0.97, the recall was 0.95, and the precision was 0.97. Additionally, a strong correlation was found between the lumen area in the ground truth mask images labeled by the authors and that in the mask images predicted by the AI (ρ = 0.97, R^2^ = 0.97, p < 0.001; Fig 4). For the test set, the accuracy of classifying vessels with severe calcification with more than a two-quadrant arc was 0.98, the recall was 0.88, and the precision was 0.97. Figs 5 and 6 show representative images predicted by our AI system.

The mean IoU and Dice scores of the validation set were 0.70 and 0.76, respectively, while those of the test set were 0.66 and 0.73, respectively (Table 1). Additionally, IoUs of the lumen area, the medial plus plaque area, calcification, and a stent in the test set were 0.86, 0.72, 0.39, and 0.05, respectively, while the Dice scores were 0.92, 0.83, 0.50, and 0.08, respectively (Table 2). When the five classes were divided into the lumen area and other classes, the IoU and Dice score of the lumen area were 0.86 and 0.92, respectively. When the five classes were divided into the medial plus plaque area and other classes, the IoU and Dice score of the medial plus plaque area were 0.66 and 0.77, respectively. The 40 IVUS images in the test set had 349 stent struts, and the recall score for detecting stent struts in each IVUS image with a stent in the test set was 0.64.

**Table 1:** Results of the validation and test sets.

	Validation	Test
Mean IoU	0.70	0.66
Mean Dice score	0.76	0.73

**Table 2:** Results for stents and each component of the vessels in the test set.

	Test			
	Lumen	Media plus plaque area	Calcification	Stent
IoU	0.86	0.72	0.39	0.05
Dice score	0.92	0.83	0.50	0.08

## Discussion

This study showed the following main findings. (1) Our AI was useful for classifying vessels with a significantly narrowed lumen area and vessels with severe calcification in IVUS images of complex lesions with a high accuracy. (2) Lumen areas in images predicted by our AI were strongly positively correlated with those in manually-labeled ground truth images. (3) Our AI accurately categorized vessel components in IVUS images of complex lesions, although not necessarily for stents. Finally, (4) although our AI detected stents in IVUS images, the IoU for stents was low.

Our AI may have the potential to classify lesions with a risk for MACEs by classifying vessels with a significantly narrowed lumen area and those with severe calcification. We showed that our AI was able to classify vessels with a lumen area less than 4 mm^2^ and those with severe calcification of more than a two-quadrant arc with a high level of accuracy (0.97 and 0.98, respectively). Moreover, the lumen area predicted by our AI system and the ground truth lumen area that was manually labeled were strongly positively correlated. A previous study reported that the minimum lumen area in IVUS images of target lesions was significantly positively related to the fractional flow reserve [23], which is an index for predicting future MACEs [24]. Additionally, the PROSPECT study showed that non-culprit vessels with a lumen area less than 4 mm^2^ had a higher risk of future MACEs than those with a larger lumen area [3]. A previous study on IVUS reported that severely calcified lesions had a tendency for a higher risk of requiring revascularization than less severely calcified lesions [25]. Another study using angiography reported that severely calcified lesions had a higher risk of MACEs than those without severe calcification [26]. Consequently, our AI might be able to classify lesions that have the most at risk of future MACEs, and this classification could contribute to improving IVUS-guided PCI by clarifying target lesions requiring treatment.

This study showed that IoUs of the lumen area and medial plus plaque area in complex lesions with significantly narrowed lumina or severe calcification were 0.86 and 0.72, respectively, for the AI. These findings suggest that the AI may also be able to classify vessel components in IVUS images of complex and simple lesions. Furthermore, IoUs of the luminal area and the medial plus plaque area were 0.86 and 0.66, respectively, when the five classes were divided into luminal area and other classes and medial plus plaque area and other classes. An IVUS study on the relationship between calcification in the culprit lesion and plaque volumes reported that the arc of calcification in vessels in target lesions was proportional to the atherosclerotic plaque burden [27]. Additionally, a serial computed tomography angiography study reported that the progression of calcification in the coronary artery was significantly positively correlated with the progression of plaque volume [28]. In many cases, PCI is performed on complex lesions (e.g., in those with severe calcification). Therefore, efficient interpretation of vessel components in complex lesions is crucial for future application of AI to pre-intervention IVUS. Previous studies have reported the use of deep neural networks for the segmentation of the lumen and media in IVUS images of simple lesions, such as those without severe calcification [12, 13]. One study reported that IoUs of the lumen area and the medial plus plaque area were 0.89 and 0.89, respectively [12], while another reported values of 0.80 and 0.81, respectively [13]. Compared with previous studies, the dataset used in our study included more calcifications and stents, which should have led to the presence of more artifacts and shadows. IVUS images with artifacts and shadows are generally more difficult to analyze and segment than those without them [13]. Under such situations, the IoU of the lumen area in our study is equivalent to that in previous studies.

Our AI had difficulty in accurately identifying stents. The mean IoU of the test set, which was calculated from the average of the IoU in each class of the test set, was only 0.66. This result was due to the low IoU (0.05) of the stents and was attributed to an insufficient segmentation of stents in vessels in IVUS images. If the AI can accurately categorize stents in vessels on IVUS images, it might be able to help cardiologists evaluate the extent of stent expansion and stent apposition, improving the outcomes of PCI. An insufficient segmentation of the stents may be a result of the small number of vessels with stents in IVUS images in the training set, and the similarity of appearance and properties of the stents to calcification in IVUS images. The proportion of IVUS images with stents in the training set was relatively small (12.3%). Additionally, because stents are small, they only comprise a limited portion of IVUS images. This situation could have led to an imbalance between the number of correct pixels of stents and the number of correct pixels of other categories, such as the lumen or medial plus plaque area in IVUS images in the training set. Moreover, stents and calcifications appear as structures with high levels of brightness and shadow [20]. Indeed, small calcifications located near the vascular surface and stents were often mistaken for each other. IVUS images of stents and areas of calcification had similar appearances and properties, and our AI might not have been able to correctly extract each feature. Ensuring more accurate segmentation of stents requires including more IVUS images with stents, using new models, such as the Pyramid Scene Parsing Network or DeepLab v3+, and using new preprocessing imaging methods [29, 30].

We consider that using U-Net is appropriate for performing semantic segmentation to improve our AI’s performance in classifying vessels with significantly narrowed lumina in IVUS images. Object recognition includes methods, such as simple image classification, object detection, and semantic segmentation, and appropriate methods need to be used for the required analysis. Simple image classification involves classifying an image into categories by recognition of objects in the image [31]. Object detection involves identifying objects in images based on their categories and identifying the location of them in the images [32]. If the classification of vessels with or without a significantly narrowed lumen is considered as a simple image classification task, it might be affected by calcification and other factors seen in IVUS images, and AI might classify vessels incorrectly. Although object detection algorithms, such as YOLO, are considered to be effective in detecting thrombus or coronary artery dissection [32], an AI using an object detection algorithm appears to be inadequate to accurately classify vessels with or without a significantly narrowed lumen. However, semantic segmentation categorizes each pixel in an image on the basis of its class or concept [11]. Therefore, by conducting segmentation of the lumen in IVUS images and calculating its area, our AI can classify vessels with a significantly narrowed lumen and other various features at a high level of accuracy.

This study has several limitations. First, the number of images in the study dataset was relatively small. Furthermore, to construct the ground truth dataset of IVUS images, manual segmentation of a large number of IVUS images was required, which was labor-intensive. We will attempt to change the learning method to semi-supervised image learning in future studies. Second, this was a single-center retrospective study, which means that potential selection bias cannot be excluded. Future studies involving a multicenter approach are warranted. Third, because expert interventional cardiologists can easily recognize and categorize IVUS images with a significantly narrowed lumen area or severe calcification in clinical practice, merely recognizing and classifying IVUS images may not be helpful for expert interventional cardiologists. An algorithm that can recognize and detect minor stent edge dissection and hematoma formation would be more useful.

## Conclusion

Our AI program is able to automatically recognize and delineate vascular structures (except for stents) in IVUS images of coronary arteries with complex lesions. Additionally, our AI can classify vessels that are likely to require treatment or special devices with a high accuracy. An AI might prove to be a powerful tool in IVUS imaging interpretation, and could promote the popularization of IVUS-guided PCI in a clinical setting.
